# NPM1/B23: A Multifunctional Chaperone in Ribosome Biogenesis and Chromatin Remodeling

**DOI:** 10.1155/2011/195209

**Published:** 2010-10-05

**Authors:** Mikael S. Lindström

**Affiliations:** Department of Oncology-Pathology, Cancer Center Karolinska, CCK R8:05, Karolinska University Hospital in Solna, 17176 Stockholm, Sweden

## Abstract

At a first glance, ribosome biogenesis and chromatin remodeling are quite different processes, but they share a common problem involving interactions between charged nucleic acids and small basic proteins that may result in unwanted intracellular aggregations. The multifunctional nuclear acidic chaperone NPM1 (B23/nucleophosmin) is active in several stages of ribosome biogenesis, chromatin remodeling, and mitosis as well as in DNA repair, replication and transcription. In addition, NPM1 plays an important role in the Myc-ARF-p53 pathway as well as in SUMO regulation. However, the relative importance of NPM1 in these processes remains unclear. Provided herein is an update on the expanding list of the diverse activities and interacting partners of NPM1. Mechanisms of NPM1 nuclear export functions of NPM1 in the nucleolus and at the mitotic spindle are discussed in relation to tumor development. It is argued that the suggested function of NPM1 as a histone chaperone could explain several, but not all, of the effects observed in cells following changes in NPM1 expression. A future challenge is to understand how NPM1 is activated, recruited, and controlled to carry out its functions.

## 1. Introduction

Ribosome biogenesis and chromatin remodeling are different processes but some parallels can be drawn. Similar to the deposition of histones onto DNA, ribosomal proteins have to bind ribosomal RNA (rRNA), and it has been assumed that both processes are prone to aggregation. With regard to size and charge, histones are reminiscent of ribosomal proteins, namely, small and basic. Histones can be divided into core histones and the H1 family of linker histones. Core histones H2A, H2B, H3, and H4 associate as H2A/H2B dimers and H3/H4 tetramers to form the histone octamer, that thus contains two H2A/H2B dimers and a H3/H4 tetramer, that the DNA is wrapped around forming a repetitive structure known as the nucleosome. Assembly of histone octamer complexes with the help of chaperones was proposed to be an ancestral feature of eukaryotes [1]. Histone chaperones are factors that bind histones and stimulate reactions involving histone transfer without being part of the final product [2]. They can be involved in histone transfer onto DNA (deposition), off DNA (eviction), from one chaperone to another, and finally histone chaperones could facilitate transfer to enzymes that use histones as substrates [2]. An emerging trend is the increasing appreciation for more active roles of histone chaperones in chromatin remodeling, for instance to increase transcription rates and facilitate replication [2, 3]. Some chaperones can store histones for a prolonged time before the actual transfer takes place and histone chaperones belonging to the nucleophosmin/nucleoplasmin family are thought to function as “storage platforms” or “sinks” [4]. 

 Some nuclear chaperones may also play a role in ribosome biogenesis by facilitating interactions between ribosomal proteins and rRNA, a function ascribed to NPM1, although solid evidence for this function is lacking at present. Nevertheless, a fraction of NPM1 is bound to preribosomal complexes and can affect the rate of ribosome synthesis. But, NPM1 is also involved in DNA replication, recombination, transcription and repair. Whereas some of the functions of NPM1 are well established, others have remained less well defined. So what are the evidences surrounding these diverse but not necessarily mutually exclusive functions of NPM1? Moreover, how does the growth promoting activities of NPM1 fit with its role as tumor suppressor? Here I provide an updated overview on the functions and interacting proteins of NPM1 while addressing some of the questions above.

## 2. Nucleoplasmin Family of Histone Chaperones

**Figure 1:**
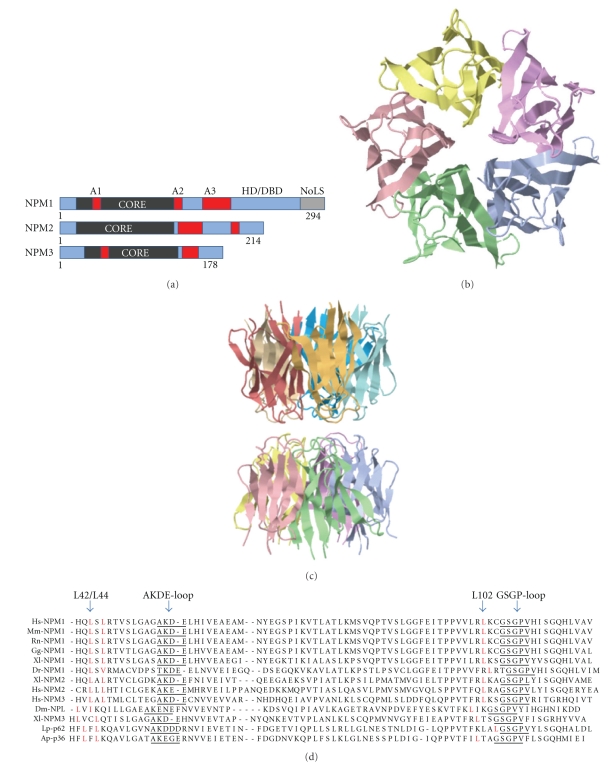
Structure of NPM1. (a) Domain representation of human NPM1, NPM2 and NPM3 proteins. Acidic patches (“A”) are indicated. (HD: heterodimerization domain, DBD: DNA binding domain, NoLS: nucleolar localization motif). (b) Pentamer structure of *Xenopus laevis* xNPM1/NO38 (PDB ID: 1XE0) as visualized in Jalview PDB viewer. (c) Two pentamers of human NPM1 (PDB ID: 2P1B) stacked in a decameric configuration. (d) Sequence alignment of the core region from different NPM1 proteins and NPM-like proteins. Clustal W was used for alignment and further editing was done with help of Chromas alignment software. The structurally important “AKDE” and “GSGP” loops are indicated in the figure as well as the location of conserved L42 and L102 residues (reference to human NPM1). Hs: Homo sapiens; Mm: Mus musculus; Rn: Rattus norvegicus; Dr: Danio rerio; Gg: Gallus gallus; Xl: Xenopus laevis; Dm: Drosophila melanogaster; Lp: Lytechinus pictus (sea urchin); Ap: Asterina pectinifera (star fish).

NPM like proteins (e.g., NPM1-NPM3) have been found in mammals, fish, birds, flies but not in bacteria or yeast (Figure 1). There are some striking differences in expression patterns, intracellular localization and functions among the different members, but one hallmark of nucleoplasmins/nucleophosmins is a well-conserved N-terminal oligomerisation domain [5]. Crystal structures of the oligomerisation domain from four members of the nucleoplasmin family have been resolved, namely, nucleoplasmin (Np) [6], *Drosophila* nucleoplasmin-like (dNLP) [7], human NPM1 [8], and *Xenopus* NO38/NPM1 [9]. The cores are in each case a pentamer, with minor differences between the different structures that could be important in terms of selectivity for binding partners (Figure 1(b)). NPM proteins can form a decamer structure by packing two pentamers on top of each other in a sandwich like structure and this structure is assumed to be involved in histone complex binding [9] (Figure 1(c)). The pentamers are not affected by DTT indicating that cysteine mediated disulphide bonds are not critical for the formation of pentamers, hence they are partially resistant during SDS-PAGE electrophoresis provided samples are not extensively boiled prior to loading [10–15]. 

Nucleoplasmin, a histone storage protein, first isolated from *Xenopus laevis* oocyte extracts [16, 17] is one of the most abundant protein in the *Xenopus* oocyte nucleus where it is complexed with the maternal histone pool [17, 18]. A nucleoplasmin pentamer may dimerize to form a decamer, competent for the association of five H2A/H2B dimers or five histone octamers, and nucleoplasmin has the ability to assemble nucleosomes *in vitro* [6]. Nucleoplasmin binds sperm nuclear basic proteins (SNBPs), including protamines, protamine-like type and histone types [19]. Nucleoplasmin promotes decondensation and remodeling of paternal chromatin following fertilization by exchanging SNBPs for histones [20]. The chromatin remodeling activity of nucleoplasmin at fertilization has been shown to be dependent on phosphorylation status [21]. Phosphorylation of nucleoplasmin enhances the H2A/H2B exchange activity during the decondensation of sperm chromatin and it also increases the ability of nucleoplasmin to promote nucleosome assembly *in vitro, *reviewed in reference [18]. Mammalian nucleoplasmin/NPM2 is expressed only in the oocyte similar to *Xenopus* nucleoplasmin. Knockout of *Npm2* in mice have no major effect on meiosis or sperm chromatin decondensation and the *Npm2^−/−^* mice are viable [22]. Nevertheless, *Npm2^−/−^*females have reduced fertility or infertility owing to preimplantation defects and nuclear abnormalities are evident in *Npm2^−/−^* oocytes, including loss of heterochromatin and changes in nucleolar organization [22]. 

 NPM3 shares many characteristics with NPM1 and NPM2 including an acidic domain and the oligomerization region, multiple potential phosphorylation sites and a nuclear localization signal [5]. *NPM3* mRNA is expressed in many human tissues with high levels in pancreas and testis [23, 24]. NPM3 protein is localized to the nucleus and nucleolus in interphase cells. Active rRNA transcription appears to be required for its nucleolar localization but on its own does not seem to bind rRNA [25]. NPM3 can bind histones [26] and affect transcription similar to NPM1, but it was shown that NPM3 lacks intrinsic histone chaperone activity *in vitro *[27]. *Npm3^−/−^* mice have not been reported to date but inhibition of NPM3 expression in mammalian oocytes prohibits paternal chromatin decondensation [28]. It is possible that NPM3 present in mice oocytes could provide a back up for the loss of NPM2, therefore the sperm chromatin remodeling function of NPM2 may not be critical in mammals, as suggested [5]. For an in-depth analysis of different NPM-like proteins and their evolution, I refer the reader to recent excellent articles on the topic [5, 29, 30].

## 3. NPM1 at a Glance

### 3.1. Gene Structure

**Figure 2:**
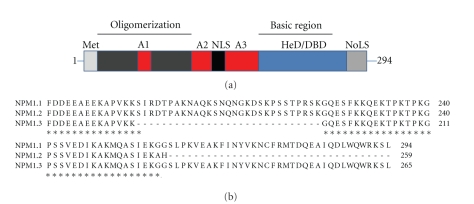
NPM1 domain organization. (a) Detailed domain representation of human NPM1 highlighting oligomerization, acidic, basic regions and where A1–A3 denotes the acidic patches (NLS: nuclear localization signal; HD: heterodimerization domain; DBD: DNA binding domain; NoLS: nucleolar localization motif). (b) Partial sequence alignment of NPM1.1 (wt) and the NPM1.2 and NPM1.3 isoforms illustrating loss of important parts of NPM1.

NPM1 (also known as nucleophosmin, NO38, numatrin or B23) is the best studied member of the NPM family and the protein is now directly linked to cancer development in humans [31]. NPM1 is highly conserved between humans, rodents, chicken and fish. Human *NPM1* is mapped to chromosome 5q35 and composed of 12 exons. Two isoforms, besides full length wt NPM1.1, have been reported, namely, NPM1.2 and NPM1.3 (Figure 2(b)). The splice variant, NPM1.2, utilizes an alternate 3′-terminal exon resulting in a shorter protein containing 259 amino acids [32]. Less is known about expression and function of the recently deposited isoform NPM1.3 but this isoform lacks an internal segment in the C-terminal region, with unknown functional significance.

### 3.2. Protein Domain Structure

NPM1 has a distinct and unique protein domain structure (Figure 2(a)) and regions required for oligomerization, chaperone activity, nucleic acids binding, nuclear localization have been described and mapped [33–35]. There are a few more interesting features in the domain organization. The first fifteen amino acids of NPM1 is a methionine rich region but the significance of this region is not clear. It might ensure strong initiation of translation since the methionine residue codons have good Kozak sequences [36] and although not an essential part of the core this methionine rich region may also affect conformation [36]. NPM1 harbors acidic stretches known as A1, A2, and A3. These patches could play a role in charge neutralization by mimicking DNA/RNA. Whereas it is evident that the NPM1 core region alone (including acidic patch A1) can bind weakly bind histones H3 and H4 the presence of the acidic A2 and A3 patches are required for histone H2A and H2B binding [37]. The C-terminal region is unique to NPM1 and contains clusters of basic amino acids providing localized positive charge followed by a highly aromatic stretch of amino acids. This region is implicated in nucleic acid binding [34, 38], ATP binding [39], histone transfer [37], ribonuclease activity [35] and nucleolar localization [40, 41].

### 3.3. Cellular Localization

NPM1 is a nucleocytoplasmic shuttling protein [42] and motifs for nuclear import as well as nuclear export have been described. The ability of NPM1 to shuttle is critical for several of its postulated functions including nuclear export of ribosomal protein L5 [43] and control of centrosome duplication [44]. NPM1 may assist small basic proteins in their transport to the nucleolus [45, 46]. Indeed, NPM1 was previously found to bind small basic viral and nucleolar proteins Rev [47], Rex [48], Tat [49] and p120 [50] and promote their nucleolar localization (Table 1). NPM1 is a predominantly nucleolar protein, however, a fraction can also be detected in the nucleoplasm. NPM1 has a dynamic localization during mitosis and is observed in nucleolar remnants, at the perichromosomal layer and at the mitotic spindle pools [51, 52]. Within the nucleolus, NPM1 is enriched in the granular component that contains maturing preribosomal particles [53]. The sequence motifs and molecular mechanisms involved in mediating nucleolar localization of NPM1 are not understood in detail but experimental evidence points out that several regions in the protein are required. Mutations in NPM1 that are predicted to disrupt both the monomer and oligomer structure markedly impair nucleolar accumulation [54]. In addition, two conserved tryptophan residues, W288 and W290, are required for NPM1 nucleolar localization, presumably by facilitating NPM1 binding to nucleic acids by providing a proper secondary structure [55]. Recently, residues K263 and K267 were shown to be functionally required for NPM1's localization to nucleoli as well as its protein stability [56, 57]. A functional nuclear localization signal is also needed [41], but some confusion in the literature exists concerning what exact motif in the central domain that is required for nuclear localization [40, 41]. NPM1.2 isoform is present in cells at low levels and is detected both in the cytoplasm and nucleoplasm further supporting the notion that the C-terminus of NPM1 contributes to nucleolar localization [58].

### 3.4. Protein Function In Vivo

Knockout of *Npm1* in mice leads to unrestricted centrosome duplication, genomic instability and impaired ribosome biogenesis [111, 112]. *Npm1^−/−^* mice display aberrant organogenesis, in particular the forebrain does not develop properly. The mice die between embryonic day E11.5 and E16.5 due to anemia resulting from severe defects in haematopoiesis [112]. However, mouse embryo fibroblasts lacking both *p53* and *Npm1* are viable and can proliferate *in vitro*, indicating that Npm1 is not strictly essential for cell growth and proliferation [111, 112]. It is also interesting that *Npm1^−/−^* embryos have a later timepoint of lethality compared to mouse embryos with ribosomal protein loss of function [113] suggesting an important but nonessential function of NPM1 in ribosome synthesis.

### 3.5. Posttranslational Modifications of NPM1

NPM1 protein is extensively modified by phosphorylation, acetylation, ubiquitylation, and SUMOylation. By studying the related protein nucleoplasmin we have learnt that phosphorylation is required for the functionality of the nucleoplasmin core domain in binding basic proteins and decondensating sperm DNA [21, 114]. From studies on nucleoplasmin we can therefore presumably learn more about NPM1 but so far, the regulation of NPM1 by phosphorylation is not well understood. NPM1 is recognized as a nucleolar phosphoprotein and several kinases, for instance casein kinase 2 (CKII) [115], polo-like kinase 2 (Plk2) [109], CDC2 [116] and cyclin E/CDK2 complex [117] can phosphorylate NPM1. Phosphorylation of NPM1 is likely to control aspects of function, localization and oligomerisation and could be related to nucleolar function and progression through mitosis [118]. Phosphorylation of NPM1 could also impinge on ribosome biogenesis by increasing NPM1's binding affinity to ribosomal components. For instance, phosphorylation by CKII enhances binding to the NLS sequences derived from the SV40 large T antigen and HIV Rev [46, 115]. 

 A flurry of papers 2008-2009 revealed an intricate series of interactions taking place between NPM1, the ARF tumor suppressor and the SUMO-pathway. Small Ubiquitin-like Modifier (SUMO) proteins are covalently attached to other proteins to modify their function in diverse cellular processes, such as apoptosis, intracellular transport, transcriptional regulation, protein stability and DNA damage repair. The SUMO-tag is removed from its protein target by SUMO-deconjugating protease (a k a sentrin-specific protease or SENP). SENP3 and SENP5 are localized within nucleoli and bind NPM1 and thus NPM1 may control the SUMO pathway [119]. Knockdown of either NPM1 or nucleolar SENPs presents with similar ribosome biogenesis defects [119]. NPM1 itself is actually SUMOylated [120] although the site(s) remains a matter of debate and SUMOylation of NPM1 may affect localization and survival pathways [121]. SENP3 can remove SUMO from NPM1 [81, 122]. Interestingly, it is now realized that the ARF tumor suppressor promotes SUMOylation of several of its interacting proteins including NPM1 [123]. Mechanistically, ARF promotes the turnover of SENP3 protein [123]. NPM1 is also ubiqutinated [54, 75, 103]. USP36 is a deubiquitylating enzyme that removes ubiquitin from NPM1 leading to stabilization of NPM1 and improved nucleolar function [84] while depletion of USP36 impairs ribosome biogenesis. Interestingly, NPM1 itself can target USP36 to nucleoli by direct binding [84]. Thus, interactions between NPM1, ubiquitin and SUMO pathways play important roles in maintaining nucleolar structure and function.

## 4. NPM1: A Dual Nucleolar Histone and Ribosome Chaperone?

NPM1 binds denatured protein substrates, a hallmark of chaperones. Indeed, one established property of NPM1 is its ability to prevent aggregation and thermal denaturation of some proteins *in vitro* [124]. Because NPM1 was found associated with preribosomes it was initially thought of as a ribosome chaperone or assembly factor. In support of this notion, a fraction of soluble NPM1 is associated with preribosomal particles, in particular 60S [43]. NPM1 facilitates cleavage of ribosomal RNA *in vitro* and acts as an endoribonuclease for the maturing rRNA transcript [13, 125]. Knockdown of NPM1 using siRNA impairs processing of prerRNA to mature 28S [75]. Moreover, blocking NPM1 nucleocytoplasmic shuttling inhibits ribosome subunit export resulting in a decrease in cell growth rate, implicating NPM1 also in preribosome export [126]. NPM1 interact directly with a subset of ribosomal proteins including RPL5 [43], RPS9 [82], and RPL23 [63]. NPM1 forms a complex with NPM3, and NPM3 was shown to negatively regulate NPM1 during ribosome biogenesis [25]. It is noteworthy that NPM1 variants lacking the nucleic-acid-binding domain also inhibit ribosome biogenesis similar to NPM3. In summary, NPM1 promotes cell growth and proliferation by supporting several distinct stages in ribosome biogenesis.

NPM1 can assemble nucleosomes *in vitro* and decondense sperm DNA similar to nucleoplasmin [37, 95], and NPM1 was suggested to be a histone chaperone in the nucleolus [9]. As mentioned NPM1 associates with rDNA and depletion of NPM1 or expression of a dominant negative NPM1 mutant lacking histone chaperone activity leads to a decrease in rDNA transcription [127], although these effects have not consistently been observed by others [111, 128, 129]. Interestingly, expression of NPM3 represses the ability of NPM1 to assemble nucleosomes *in vitro *[27], similar to the negative regulation of NPM1 function by NPM3 seen during ribosome biogenesis. Is it possible that these regulatory functions are two sides of the same coin? 

 Identification of the nucleolar p14ARF/p19Arf (henceforth ARF) tumor suppressor as a major NPM1 binding partner ignited interest in the function of NPM1 in the nucleolus [75, 128, 130]. Enforced expression of ARF blocks NPM1 nucleocytoplasmic shuttling [128], increases degradation of NPM1 protein and reduces 28S rRNA processing [75]. Under normal conditions, NPM1 promotes the nucleolar localization and stability of ARF, but whether NPM1 also can block ARF functions remains controversial [131, 132]. It was argued that under normal conditions NPM1 mainly act to promote nucleolar localization of ARF [133]. Interestingly, other groups have found that ARF reduces protein synthesis by inhibiting polysome formation [129], and that ARF binds to rDNA to interfere with transcription [134]. These are interesting findings given that NPM1 binds to the rDNA gene promoter and plays a role in ribosome nuclear export. However, ARF binds to other targets than NPM1 in the nucleolus [130] and it is therefore unlikely that all the effects of ARF is funneled through NPM1. One problem in these types of studies are possible indirect effects stemming from activation of stress responses, such as p53 induced cell cycle arrest, that in some settings could complicate the interpretation of cell growth data (“the chicken and the egg problem”). A second problem could involve incomplete NPM1 knockdown in those experiments that rely on RNAi given that NPM1 is an abundant and stable protein.

## 5. NPM1 Functions in DNA Replication, Transcription and Repair

NPM1 has been implicated in the processes of DNA replication, recombination, transcription and repair, reviewed in [31]. And, as mentioned, NPM1 interacts with histones H3, H4, H2A and H2B. NPM1 may participate in chromatin remodeling related events by affecting the assembly of nucleosomes, or regulate the modification of histones through the recruitment of histone modifying enzymes. Interestingly, nucleolin (C23), a major nucleolar protein has also been implicated in DNA replication and transcription in analogy with NPM1 [135], although nucleolin and NPM1 have no structural similarity.

### 5.1. DNA Replication

NPM1 binds retinoblastoma protein (pRB) and synergistically stimulates DNA polymerase alpha activity *in vitro* and may therefore influence DNA replication processes [77, 136]. In addition, NPM1 can also stimulate *in vitro* replication of adenoviral DNA [95]. Another example of a histone chaperone involved in replication is ASF1 that preferentially localizes to active DNA replication forks [137].

### 5.2. DNA Transcription

NPM1 has been directly implicated in gene transcription in several ways. First, NPM1 interacts with c-Myc through binding at c-Myc promoters facilitating RNA pol II driven transcription [59]. NPM1 regulates Myc protein turnover as well and may therefore directly impact on cell growth and transformation during tumor development [105]. Second, NPM1 can interact with HEXIM1, a regulator of RNA pol II transcription, to facilitate transcription [65]. Third, combination of acetylation on core histones and NPM1 itself enhances transcription rates [138]. Acetylation of NPM1 (Ac-NPM1) leads to disruption of the nucleosome and transcriptional activation. This indicates a novel and more active role of NPM1 in chromatin remodeling [138]. Ac-NPM1 is preferentially found in the nucleoplasm in association with RNA polymerase II and the increase in Ac-NPM1 seen in cancer could be of mechanistic importance [139]. On the other hand, NPM1 binds GCN5 during mitosis to inhibit GCN5 mediated acetylation of free and mono-nucleosomal histones [70]. Fourth, NPM1 act as a corepressor or coactivator of transcription by binding to YY1, IRF1, p53, NF-*κ*B and other transcription factors (Table 1). For example, it was shown that NPM1 participates in the transcriptional response to retinoic acid in myeloid cells [64]. During retinoic acid induced differentiation, NPM1 forms a complex with activating protein transcription factor 2*α*, and performs a corepressor function through recruitment of histone deacetylases [64]. Fifth, and of particular interest, are the findings that NPM1 is involved in the regulation of RNA pol I transcription in the nucleolus [127], and is critical in mediating induction of rDNA transcription factor TAF(I)48 [140]. Modulation of RNA pol I transcription could be a critical activity of NPM1 because changes in rDNA transcription is an important molecular alteration in cancer cells [141]. RNA pol I activity is tightly controlled by several tumor suppressors and oncogenes such as p53 and c-Myc, respectively [142]. Because both c-Myc and NPM1 bind to nucleolar rDNA chromatin and can stimulate RNA pol I transcription [127, 143, 144], it is likely that overexpression of NPM1 facilitates c-Myc driven rRNA synthesis, similar to how NPM1 facilitate c-Myc function at RNA pol II controlled promoters. Such a mechanism could be relevant to consider when thinking about how NPM1 contributes to cell growth and oncogenic transformation. Binding of NPM1 to nucleolar chromatin requires, somewhat surprisingly, the RNA binding activity of NPM1 and indirectly the nucleolar transcription factor UBF [145]. Moreover, NPM1 facilitates nucleolar localization of the RNA polymerase I transcription termination factor (TTF-1) [87]. In summary, several lines of evidence point toward a supporting role for NPM1 in RNA pol I and II transcription.

### 5.3. DNA Repair

DNA strand breaks trigger activation of cellular pathways resulting in a DNA damage response and DNA repair. A DNA strand break results in global and local changes in chromatin together with activation and recruitment of several repair and signaling factors. Upon sensing DNA damage, or in the initiation of DNA repair itself, NPM1 translocates from the nucleolus to the nucleoplasm and binds chromatin [146]. In particular a pool of phosphorylated NPM1 is recruited to IR-induced DNA damage foci [147]. Inhibition of rDNA transcription or rRNA processing, in the absence of DNA damage, also often provokes rapid translocation of the otherwise predominantly nucleolar NPM1 protein to the nucleoplasm [148, 149] and by this change in localization NPM1 may then functionally interact with different cellular pathways. It is interesting that NPM1 mRNA and protein was found to be significantly induced following UV-induced DNA damage [150] and coupled with an increase in NPM1 expression there was enhanced RNA binding activity [151]. Enforced NPM1 expression in turn renders cells more resistant to UV-induced cell death [152]. It is conceivable that NPM1 could function as a histone chaperone under, or following, repair of DNA strand breaks. One example of a histone chaperone with a similar function already established is yeast CAF1 that mediates histone H3-H4 assembly during nucleotide excision repair. CAF1 is critical in chromatin assembly during double strand break repair but is not strictly needed in the repair process itself [153]. It would therefore be relevant to investigate if the NPM1 mediated prosurvival effect can be explained by a histone chaperone function active in the DNA repair process.

## 6. What Is the Function of NPM1 at the Mitotic Spindle?

The mitotic spindle includes the spindle microtubules, an array of associated proteins, and the metaphase centrosomes also present at the spindle poles. At the spindle poles, microtubules are nucleated by the centrosomes while the spindle fibers are the structures that separate the chromosomes into the daughter cells during cell division. Failure of spindle assembly can lead to spindle checkpoint activation and if not properly regulated may result in aneuploidy. *Npm1* heterozygosity in mice led to unrestricted centrosome duplication and genomic instability [111, 112]. Interestingly, a fraction of NPM1 protein is detected at the mitotic spindles pool during metaphase, easily detected using conventional immunofluorescence microscopy [52]. It should be pointed out that there are other nucleolar proteins at the mitotic spindles such as nucleolin and fibrillarin, meaning that NPM1 being a nucleolar protein at the spindle is not in a unique position. At the spindles, NPM1 colocalizes with NuMA (nuclear mitotic apparatus protein) [52]. The spindle pool bound form of NPM1 is modified, presumably phosphorylated, as indicated by migration shifts in immunoblots [52]. NPM1 may play a more direct role in centrosome duplication in some cells given evidence that NPM1 associates with the unduplicated centrosome in interphase and is released by cdk2/cyclin E mediated phosphorylation on NPM1 residue Thr199 leading to initiation of centrosome duplication [117]. Other investigators have not been able to detect NPM1 at the centrosome in interphase cells using proteomics or immunofluorescence [154], although it should be taken into consideration that the immunogenic epitopes and conformation of centrosome bound NPM1 could be different [155]. In mice, phosphorylation of Npm1 Thr198 was shown to occur throughout the cell cycle and the cell growth and proliferation rates were similar to wt NPM1. This suggests that phosphorylation of Npm1 Thr198 is not a critical event for normal cell proliferation in mice [156]. 

 Centromeres (not to be confused with centrosomes) are the chromosomal domains that control the mitotic behavior of chromosomes. NPM1 also associates with a CENPA (Centromere protein A) containing complex [157]. CENPA encodes a histone H3 related histone fold domain and CENPA is a presumed component of a modified nucleosome-like structure in which it replaces histone H3. The finding of NPM1 in complex with CENPA implicates NPM1 also in centromere control. Recent studies confirmed NPM1 in these complexes but did not find any evidence for a major function of NPM1, although knockdown of NPM1 was not complete in some experiments [158, 159]. Nevertheless, in rapidly growing HeLa cells, the depletion of NPM1 resulted in mitotic arrest due to spindle checkpoint activation and p53 induction and these cells showed signs of defects in mitotic spindle and centrosome formation [160]. Data taken together suggest a supporting histone chaperone function mediated by NPM1 involving centromeric chromatin. Interestingly, depletion of nucleolin results in growth arrest, increased apoptosis and numerous nuclear alterations, multipolar spindles together with defects in centrosome duplication [161] very similar to the inactivation of NPM1. What makes nucleolin interesting in this context is that it also functions as a histone chaperone [162]. To understand the dual function and localization of NPM1, as well as nucleolin, remains a challenge.

## 7. NPM1 and Cancer

NPM1 is clearly having both growth promoting and tumor suppressive functions. Overexpression of NPM1 enhances cell division and cell growth presumably through stimulatory effects on rDNA transcription, ribosome subunit export and DNA replication during S-phase. A transforming role for NPM1 was observed in NIH3T3 cells [68] and overexpression of NPM1 could possibly promote oncogenesis by interfering with the activation of p53 by ARF. Similar to oncogenes, such as Ras, it was found that NPM1 overexpression induces cellular senescence in human fibroblasts [74]. The level of NPM1 is generally elevated in tumor cells compared with normal cells. Increased levels of NPM1 may at least in part reflect a higher growth rate and an increased demand for ribosome biogenesis in cancer cells [163] and NPM1 is induced when B-cells, T-cells and Swiss 3T3 cells are stimulated with various mitotic agents [164–166]. NPM1 is overexpressed in many types of major human solid tumors including, but not limited to, tumors of the thyroid [167], brain [168], liver [169] and prostate [62]. *NPM1* has been shown to be a target of oncogenic Myc and *NPM1* mRNA can be directly induced by Myc through binding to the *NPM1* promoter [170]. The ability of NPM1 to suppress apoptosis and to promote DNA repair may play a significant prosurvival role during tumor development. There is however no solid evidence yet demonstrating that (wt) *NPM1* is acting as a *bona fide* proto-oncogene* in vivo, *when overexpressed. 

 Disruption of the *NPM1* gene by translocation or heterozygous deletion is found in human hematopoietic malignancies [171], such as acute promyelocytic leukemia (APL), anaplastic large cell lymphoma (ALCL), and in premalignant myelodysplastic syndromes (MDS), reviewed in [31, 171]. Interestingly, translocations occur in the NPM1 N-terminal region where it fuses to different partner genes, such as anaplastic lymphoma kinase (ALK), retinoic acid receptor *α* (RAR*α*), or myeloid leukemia factor 1 (MLF1) in ALCL, APL and MDS respectively. In these malignancies, NPM1 contributes to tumor development by activating the oncogenic potential of the fused protein partner, that is, ALK, RAR*α*, or MLF1 [172, 173]. Furthermore, loss of one allele of *NPM1* is frequently observed in patients with *de novo* and therapy-related MDS [31]. The significance of *NPM1* as a cancer gene was somewhat dubious because it was thought that the NPM1 part (of the fusion protein) was only used an essential dimerization interface. For instance, in the case of NPM1-ALK, the transforming capacity correlates with an ability to form oligomers and NPM1-ALK unable to form oligomers (dimerize) is nontumorigenic [172]. This unclear role of NPM1 would change however, as it in 2005 was found that one-third of adult acute myeloid leukemia (AML) cases display aberrant cytoplasmic expression of NPM1 with mutations occurring in the exon-12 of *NPM1*, therefore directly implicating *NPM1* as a cancer gene [174]. Analysis and clinical studies regarding NPM1c+ AML has recently been reviewed extensively and will not be covered further [175].

## 8. NPM1 as a Tumor Suppressor

NPM1 is acting as a haploinsufficient tumor suppressor in blood cells [176]. First, loss of NPM1 destabilizes the ARF protein that occurs in parallel with a failure of ARF to properly localize to nucleoli [111]. Decreased levels of ARF means increased rate of oncogenic transformation of mouse fibroblasts because ARF is critical to activate p53 in response to oncogene activation in mice [111, 177]. The notion that NPM1 is a critical caretaker of ARF is also supported by the fact that ARF is delocalized and functionally handicapped in cells expressing AML derived oncogenic NPM1c mutants [178, 179]. It should be mentioned that NPM1 regulates nucleolar localization of Fbw7*γ* isoform and that NPM1c has lost also this function leading to increased levels of MYC [105], given that MYC degradation requires Fbw7*γ* [180]. The intricate dynamics of the ARF-NPM1-Myc network have been reviewed elsewhere in greater detail [181]. Second, *NPM1 *heterozygosity results in genomic instability as shown by aneuploidy, increased centrosome numbers and DNA damage checkpoint activation that in a secondary stage enhances the rate of oncogenic transformation both *in vitro *and *in vivo*. In fact, *Npm1^+/−^* mice often develop hematological malignancies following on a condition resembling myelodysplasia [112]. 

Disturbances in nucleolar or ribosomal function are known as nucleolar stress or ribosomal stress and are often associated with p53 tumor suppressor pathway activation [182]. Defects in the ribosome biogenesis machinery often presents with bone marrow failure and increased cancer risk. One notable syndrome is Diamond-Blackfan anemia (DBA) caused by mutations in ribosomal proteins (*RPS19, RPL11, RPL5* and others) leading to defective 40S or 60S ribosomal subunit production with elevated risk of leukemia and osteosarcoma [183]. As mentioned earlier, inactivation of nucleolar proteins NPM1 and nucleolin also leads to mitotic spindle defects, genomic instability and accumulation of DNA damage that in turn activates p53. The extent of genomic instability in DBA patients is not clear. The point being, it is not entirely clear if reducing levels of NPM1 activates a DNA damage response, a nucleolar stress response, or both and it could be of relevance to further investigate this. 

 In summary, loss of one allele of *NPM1* is one step closer to cancer but it is also worth noting that NPM1 has *not* been shown to repress genes involved in cell cycle progression, induce apoptosis in response to cell damage, or to induce cell cycle arrest following DNA damage. Therefore, *NPM1* is not your “typical” tumor suppressor gene. Rather, NPM1 acts as a context dependent tumor suppressor, that is downstream of the cell cycle machinery and expression level and localization of the protein is of major importance.

## 9. Mechanisms of NPM1 Nuclear Export

Discovery of the NPM1c mutant in AML, which is a combined gain-of-function (nuclear export) and loss of function (nucleolar localization) mutant, represents a fundamental breakthrough in understanding the molecular pathogenesis of a subset of AML cases [174]. NPM1c utilizes a Crm1-mediated nuclear export mechanism through a frameshift mutation resulting in the creation of a functional nuclear export signal (NES) that combined with other amino acid substitutions is working to prevent NPM1c nucleolar localization [184]. Related to the work on NPM1c, has been the parallel hunt for endogenous NES in NPM1 and two groups have found different candidate NES within the NPM1 oligomerization domain. One motif is the 42-LSLRTVSL-49 sequence in hNPM1, wherein mutations L42A and L44A block NPM1 nuclear export [43]. Similarly, introducing the L102A mutation within the second NES 94-ITPPVVLRL-102 motif blocks NPM1 nuclear export [44] and interestingly, introducing the L102A mutant also prevents NPM1c shuttling. An alternative expanded model would take into consideration the structure of NPM1 core. Presumably, mutations introduced in critical loop residues within the oligomerization domain leads to a collapse and a failure to maintain the proper structure of the monomer [54]. Other mutations could impair the conformation or disrupt the hydrophobic thermostable core of NPM1 while leaving the monomeric structure relatively unaffected. Residues L102, L42 and L44 (reference to human NPM1) are all highly conserved residues in the entire nucleoplasmin family (Figure 1(d)). Conceivably, some NPM1 core mutants that have lost structural and/or functional properties will likely also affect nuclear export. In other words, NPM1 mutants with structural defects might fail to undergo export depending on the association with other cellular factors in combination with conformational changes. This model can easily be tested by further mutagenesis of conserved residues within the NPM1 core. The availability of the NPM1 core structure allows also for *in silico* prediction of mutations in terms of structural effects to some extent [54]. Hence, the analysis of NPM1 nuclear export should take structural effects into consideration and more remains to be discovered about NPM1 nuclear export.

## 10. Targeting NPM1 in Cancer

Under the assumption that high levels of NPM1 makes cancer cells addicted to it, knocking down NPM1 using RNAi or small molecule inhibitors could block cell growth. Inhibition of NPM1 in cancer cells but not in normal cells is therefore an attractive, but still theoretical, therapeutic approach. Retroviral HIV encoded Rev is a small basic and cytotoxic protein with a predominantly nucleolar localization, similar to ARF. A Rev derived peptide that binds to NPM1 with high affinity interferes with NPM1 function. The authors showed that NPM1's ability to transactivate a PCNA promoter construct was impaired by this Rev peptide [185]. Another peptide is CIGB-300 (P15-TAT), an anticancer peptide that can bind NPM1 and prevent CK2 driven phosphorylation leading to nucleolar breakdown and cancer cell apoptosis [186]. An interesting nonpeptide compound is avrainvillamide, an alkaloid, that targets residue C275 in human NPM1 and also induces p53 activity in cells [187]. 

 What about small molecules that inhibit NPM1 oligomerization? The compound NSC348884 is a putative NPM1 small molecule inhibitor that disturbs NPM1 oligomer formation and induces p53 that is followed by apoptosis in cancer cells [188]. NSC348884 acts by disrupting a hydrophobic pocket required for oligomerization. In a similar study to indentify inhibitors of NPM1, Jian et al. used a SELEX to search for RNA aptamers that bind NPM1 [189]. They identified a number of RNA aptamers that bound the central region of NPM1 resulting in disturbed NPM1 oligomerization, nucleolar delocalization and increased sensitivity to apoptosis [189]. Studies in mice indicates a tumor suppressor role for NPM1 as discussed and therefore more studies are needed in order to clarify the potential usefulness of the above mentioned strategy considering then the complexity of NPM1.

## 11. Other Functions of NPM1

Undoubtedly NPM1 is a multifunctional protein that has found its way into most chapters in the cell biology text book. NPM1 was recently implicated in neural and hematopoietic stem cell fitness [190, 191], mRNA splicing and stability [192, 193], and NPM1 may even act as an alarmin in the immune system [194]. NPM1 was also found to be a nuclear PIP3 receptor, and the PIP3-NPM1 complex mediates the antiapoptotic effect of neural growth factor (NGF) through inhibition of caspase activated DNase [98]. There are several other NPM1 associated proteins than those enlisted in Table 1, but that remains less well characterized. In proteomics studies for instance, NPM1 was found associated with numerous ribosomal proteins and RNA/DNA helicases [82]. In addition, NPM1 was earlier found to associate with CDC14 (involved in centrosome organization), SNF2 (chromatin remodeling), Nup98 (nuclear pore) and CENPF (centromere protein F) [195]. 

 There are also two interesting activities of nucleoplasmin that could be related to NPM1. First, nucleoplasmin can decondense chromatin in undifferentiated F9 mouse cells through epigenetic changes that involves histone modifications and release of heterochromatin proteins [196]. Through this function, nucleoplasmin enhances activation of oocyte specific genes in these mouse cell nuclei when nuclei are injected into frog eggs [196]. Second, apoptotic chromatin condensation can be regulated by nucleoplasmin [197]. Both the reprogramming and the apoptotic functions of nucleoplasmin are very interesting and it would be significant to investigate if NPM1 could have related functions.

## 12. Conclusions

**Figure 3:**
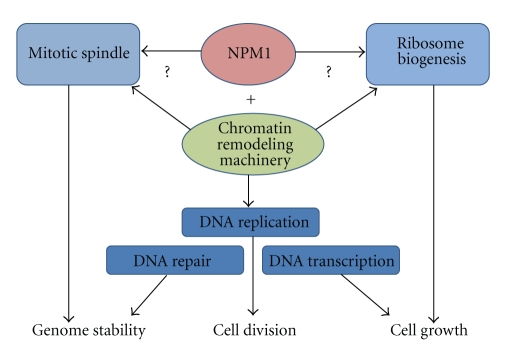
NPM1 is a multifunctional histone binding protein. NPM1 can affect DNA replication, repair and transcription by interacting with the components of chromatin such as histones and chromatin remodeling proteins. NPM1 is also required for a controlled progression through mitosis and NPM1 can promote ribosome biogenesis. These effects may arise through the ability of NPM1 to bind histones at the centromeres or in the nucleolus, respectively.

As enlisted in this paper, NPM1 participates in a number of cellular processes including multiple aspects of gene expression and certain modified forms of NPM1 seem to play an active in role in RNA pol II transcription. NPM1 also interacts with a number of proteins at the mitotic spindle as well as in the nucleolus. A histone chaperone function provided by NPM1 is presumed to be of importance at each cellular location but exactly how NPM1 facilitates transcription, replication and repair remains elusive. The main function of NPM1 at a given moment could also be determined by the cell cycle stage. Hence, NPM1 may preferentially promote ribosome biogenesis in G1, facilitate DNA replication during S-phase while supporting chromosome segregation in mitosis (Figure 3). What we now have to better understand is how NPM1 is recruited and controlled at these different “activity stations” in the cell. In this regard, the recent finding that phosphorylated NPM1 (T199-p) is specifically recruited to DNA damage sites is particularly interesting [147]. 

 Ample amounts of data show how NPM1 participates in multiple steps in ribosome synthesis, but it is not easy to understand how NPM1 can modulate rDNA transcription, rRNA processing and export of the ribosome at the same time. Because these processes most likely are coupled, but unclear how, it will not be straightforward to dissect the functional role of NPM1 at each stage of ribosome biogenesis. Of course, a function of NPM1 on nucleolar chromatin does not exclude strictly separate later-stage functions in ribosome assembly and export given that NPM1 binds both RNA and ribosomal proteins. The same problem have surrounded nucleolin, being implicated in several ribosome biogenesis steps (rDNA transcription, rRNA processing and assembly) and associated with histone chaperone properties [162]. It now appears that one of the key roles of nucleolin is to permit transcription of rDNA genes [135]. Further studies on the role of NPM1 in ribosome biogenesis are needed to clarify its function. Especially, how NPM1 could contribute to cell growth and transformation by cooperating with oncogenes in the regulation of rDNA chromatin deserves more attention. We also need to obtain a better understanding of the general mechanisms of rDNA transcription and rRNA processing in human cells. Such efforts are in part hampered by current limitations in relevant assays to measure and monitor loading of ribosomal proteins onto RNA and the kinetics of rRNA processing.

## Figures and Tables

**Table 1:** NPM1 binding proteins.

*DNA replication, transcription, and repair*
MYC [59]; APE1/Ref-1 [60]; NFKB1 [61]; AR [62]; MIZ1 [63]; AP2*α* [64]; HEXIM1 [65]; YY1 [66]; CBF-A [67]; IRF1 [68];
MNDA [69]; GCN5 [70]; Histones [37]; C/EBP*α* [71]; Tpt1 [72]; DOT1L [73]

*Cell cycle control *
p53 [74]; ARF [75]; MDM2 [76]; pRB [77]; p21 [78]; GADD45A [79]

*Ribosome biogenesis *
EBP1 [80]; SENP3 and SENP5 [81]; RPL5 [43]; RPS9 [82]; RPL23 [63]; Nucleolin [83]; p120 [50]; NPM3 [25]; USP36 [84];
Nucleostemin [85]; PES1 [86]; TTF1 [87]; FRGY2a/YB1 [88]; NSUN2 [89]

*Viral replication *
Human T-cell lymphotropic virus Rex [48]
HIV Rev and Tat proteins [47]
Hepatitis delta virus antigens [90]
Hepatitis B virus core protein [91]
Mouse mammary tumor virus p14 env-related protein [92]
Hepatitis C virus core [93]
Japanese encephalitis virus core protein [94]
Adenovirus basic core protein [95]

*Apoptosis*
Bax [96]; PARP-1 and PARP-2 [97]; PIP3 [98]; GAGE [99]

*Stability and splicing of mRNA*
hnRNPU [100]; hnRNPA1 [100]; NSP 5a3a [101]

*Protein modification, synthesis, and degradation*
PKR [102]; BRCA1-BARD1 [103]; AKT [104]; Fbw7*γ* [105]; HLJ1 [106]

*Mitotic spindle, cytoskeleton, and centromeres *
CRM1 [44]; RPGR and RPGRIP1 [107]; Eg5 [108]; Plk2 [109]; CTCF [110]
